# Anthocyanins from *Cornus kousa* ethanolic extract attenuate obesity in association with anti-angiogenic activities in 3T3-L1 cells by down-regulating adipogeneses and lipogenesis

**DOI:** 10.1371/journal.pone.0208556

**Published:** 2018-12-06

**Authors:** Muhammad Imran Khan, Jin Hyuk Shin, Tai Sun Shin, Min Yong Kim, Nam Jun Cho, Jong Deog Kim

**Affiliations:** 1 Department of Biotechnology, Chonnam National University, Dun-Duk Dong, Yeosu, Chonnam, Korea; 2 Department of Food Science and Nutrition, Chonnam National University, Yongbong-ro, Buk-gu, Gwangju, Republic of Korea; 3 Department of Refrigeration Engineering, Chonnam National University, Dun-Duk Dong, Yeosu, Chonnam, Republic of Korea; Universidad Pablo de Olavide, SPAIN

## Abstract

*Cornus kousa* the Korean dogwood has been traditionally used in East Asia as therapeutic traditional medicine however biological activities of *Cornus kousa* have not been investigated previously. The aim of the present study was to evaluate anti-obesity activities coupled with anti-angiogenic activities of anthocyanins rich fraction of ethanolic leaf extract of *Cornus kousa* (ELECk) in HUVECs and 3T3- L1 cells. Dried plants leaves were extracted with 70% ethanol and anthocyanin fraction (AnT Fr) was obtained by eluting the ethanolic extract through non-polar macroporous resin and further purification by HPLC. Antiangiogenic activities were determined by antiproliferative effect of AnT Fr on HUVECs. In the presence of various concentrations of AnT Fr, 3T3-L1 preadipocytes were induced to differentiate. Lipid accumulation in differentiated adipocytes were quantified by Oil-Red O staining. AnT Fr significantly suppressed angiogenesis by inhibiting proliferation and tube formation of HUVECs via downregulating VEGRF 2, PI3K, β‐catenin, NF‐kB, and Akt1 in a dose dependent manner. AnT Fr inhibited lipid accumulation by down-regulating adipogenesis and lipogenesis promoting signaling proteins, PPARγ, CCAAT, C/EBPα, aP2, FAS, and LPL, however enhanced AMPK activation to p-AMPK in 3T3 cells quantified and expressed by western blotting. AnT Fr inhibit lipid accumulation by regulating adipogenesis and lipogenesis related genes and signaling proteins. The anti-obesity activities exerted by *Cornus kousa* are associated with antiangiogenic activities of anthocyanins rich fraction of *Cornus kousa*. Hence the presence of bioactive anthocyanins, *Cornus kosa*, is a good candidate for nutraceutical and pharmaceutical formulation for treating or controlling obesity.

## Introduction

Obesity is a metabolic disorder and inflammatory disease that affected over 600 million people worldwide. Clinical obesity is defined when body mass index (BMI) ≥ 30 kg/m^2^ [1]. Obesity is caused by excess accumulation of triglycerides (TG) in adipose tissue due to an imbalance of energy intake and expenditure [2]. Obesity promotes development of other metabolic disorders such as diabetes and cardiovascular diseases as well as chronic diseases such as stroke, osteoarthritis, sleep apnea, cancer, and inflammation [3, 4]. Obesity is a high-risk factor for different types of cancers, including pancreatic cancer, hepatocellular carcinoma, prostate cancer, gastric cancer, and breast Cancer [5–7]. Obese adipose tissue produces inflammatory cytokines and mediators that can promote tumor metastasis and lead to cancer [8,9]. Lipogenesis and adipogeneses are main processes involved in the production and accumulation of lipids inside adipose tissues. They are promoted by various genes, transcriptional factors, and signaling molecules such as fatty acid synthase (FAS), fatty acid binding protein (aP2), stearoyl-CoA desaturase-1 (SCD-1), lipoprotein lipase (LPL), CCAAT/enhancer-binding protein α (C/EBPα), peroxisome proliferator-activated receptor γ (PPARγ), and sterol regulatory element binding protein-1c (SREBP-1c) [10–12]. Hence, these signaling molecules, transcriptional factors, and enzymes are major targets for controlling and treating obesity. Activated monophosphate kinase (AMPK) also has a strong contribution to lipid metabolism and adipogenesis. However, phospho-AMPK (p-AMPK) is inversely related to lipid synthesis and adipogenesis. Angiogenesis is also associated with obesity by promoting the expansion and growth of adipose tissues. Angiogenesis is the physiological process through which new blood vessels are formed from existing ones. It is the most important process that provides cells satisfactory supply of nutrients and oxygen [13]. It is a common phenomenon under normal physiological conditions, i.e., development of embryo, wound healing, and reproduction. However, pathological angiogenesis causes neovascularization for tumor growth and metastasis, leading to angiogenesis dependent cancer and autoimmune disease. Malignant tumors can continue to grow by receiving nutrients using blood vessels around them. These cancer cells can be connected to the circulatory system and transferred to other parts such as lungs and liver.

Understanding the mechanism of obesity, especially the role of inflammation and angiogenesis in fat mass expansion, can lead to therapeutic approaches for controlling obesity and its related disorders. Adipose tissue is enclosed by one or more capillaries that provide nutrients and supply oxygen for its growth and expansion [14]. Adipose tissue produces several angiogenic factors such as leptin, angiostatin, angiopoietin-2, and endostatin. However, angiogenic activity is mainly related to vascular endothelial growth factor (VEGF/VEGFR) [15–16]. Controlling angiogenesis will cause inhibition of growth and expansion of adipose tissues, thus controlling obesity. To overcome side effects associated with currently available anti-obesity medicines and obtain better prevention and treatment options, medicines with minimum or zero side effects are needed [17]. Using natural products with bioactive nutrients has been proposed as an effective solution to combat and even prevent obesity [18]. This can be a great alternative strategy for developing effective and safe anti-obesity medications in the future. A variety of natural products, including crude extracts and pure natural compounds, can cause weight loss and prevent dietary obesity. Various plant products such as saponins, polyphenols, flavones, and caffeine have been reported to possess anti-obesity activities [19,20].

*Cornus kousa* (Korean dogwood, Japanese dogwood) belongs to family Cornaceae. It is a native plant to East Asia, including Korea, China, and Japan. *Cornus kousa* has been traditionally used in East Asia as edible fruits and therapeutic medicine. Some studies have shown that *C*. *kousa* has compounds such as anthocyanins and flavonoids with strong antioxidant activities [21]. However, biological activities of *Cornus kousa* are poorly described. The objective of the present study was to examine antiangiogenic effects and anti-obesity activities of anthocyanins present in *Cornus kousa* ethanolic leaf extract.

## Materials and methods

### Preparation of plant extracts

Plant materials used in this study were bought from a local food shop located in the open food and vegetables market of Yeosu (Dun-Duk Dong, Yeosu, Chonnam, 550–749, Korea) The plants materials were consisted of leaves (Turned red because of accumulation of anthocyanins). It was freshly harvested in autumn from a farm (Myeongin Shin Kwang-Soo's tea farm Suncheon, Jeonnam chonnam republic of Korea) and were packed in polyethene bags (1 Kg/ bag) in natural form and have not undergone any pre-treatment or processing. The plant is well known and native to Korea that’s why plant materials were easily identified by Human Herb Research Center (Korea). It was washed thoroughly with distilled water, dried, and ground into pomace powder. The powder (500 g) was then extracted with 70% ethanol by refluxing in heating mantles (Global Lab GLHMP-F500) at 60°C for 8 hours. The resulting extract was filtered with membrane filtration system. The filtrate was then concentrated with a rotary vacuum evaporator (EYELA N-1000) at 50-60°C. Concentrated extracts were dried by lyophilization. After storing portions of the extract at -20°C, the rest was subjected to further purification with non-polar macroporous resin (D101), first eluted with 0.3N NaoH and then eluted with 100% ethanol. The fraction obtained was purified further by HPLC and was subjected to GC-Ms for analysis and quantification of the present anthocyanins compounds.

### Antiangiogenetic assays

To determine the anti-angiogenic activity of anthocyanins rich fraction (AnT Fr) of ethanolic leaf extract of *Cornus kousa* (ELECk), first toxicity of AnT Fr was checked by MTT (dimethyl thiazol diphenyl tetrazolium bromide) assay. After determining the safe doses(concentrations), anti-angiogenic activities were determined by its inhibitory potential on HUVECs (Humen umbilical vein endothelia cell) proliferation and tube formation.

#### Cell viability essay

Stock solution of AnT Fr was prepared as 1mg/ml and then diluted to various concentrations. Human umbilical vein endothelial cell (HUVEC) line was purchased from the Korean Cell Line Bank (Seoul, Korea). HUVECs were cultured in EBM-2 media (Clonetics, USA) supplemented with EGM-2 Single Quot Kit (Clonetics, USA) at 37°C in a humidified 5% CO_2_ incubator. Cell viability was determined by MTT assay. HUVECs (1 × 10^4^ cells/well) were seeded into a 96-well plate, incubated for 24 h, and then treated with various concentrations (5, 10,15, 20,25,30,50,100, and 200 μg/ml) of AnT Fr for another 24 h at 37°C in a humidified 5% CO_2_ incubator. Control groups of HUVECs were grown in the absence of AnT Fr. Then 0.5% MTT solution (Sigma, MO, USA) was added to the medium and incubated for 4 h at 37°C. MTT medium was removed by aspiration and DMSO (Sigma, MO, USA) was added to each well and keep for 15 min to dissolve formazan crystals. Absorbance was measured at wavelength of 540 nm using a microplate reader (Biochrom Ltd., Cambridge, UK). The experiments were run in triplicate.

#### Anti-angiogenic activities/inhibition of tubes formation

To determine anti-angiogenic activities of AnT Fr, 24-well culture plates were used for this assay. They were coated with Matrigel (BD Bioscience, MA, US) at 150 μl/well that was then allowed to solidify at 37°C for 1 hr. HUVECs suspended in medium (2.5 × 10^4^ cells/well) were added to Matrigel-coated wells and incubated at 37°C for 4 h. Various concentrations (5, 20, 50, and100 μg/ml) of AnT Fr were then added to wells and incubated at 37°C for 4 h. Tube formation was observed and photographed using a phase contrast inverted microscope (Nikon, Tokyo, Japan). Tube lengths in five photographs were obtained from random fields in each well. They were analyzed using Scion Image software (NIH, ML, USA). The experiment of the inhibitory effects of AnT Fr on angiogenesis were performed more than 2 times.

#### Analysis of gene expression level

To elucidate the effect of AnT Fr on the expression of angiogenic proteins (signaling molecules), RT- PCR analysis was performed using gene-specific primers. Expression (mRNA) levels of angiogenesis supporting signaling proteins in HUVECs were quantified to determine the effect of AnT Fr on angiogenesis. HUVECs were cultured in EBM2 media supplemented with 0.3% FBS in 6-well plate and incubated at 37C in CO_2_ incubator. Cells were then treated with different concentrations (5, 20, 50, and 100 μg/ml) of AnT Fr and incubated in the CO_2_ incubator for 24 hours. Total RNA was isolated from HUVECs using TRI reagent (Sigma Aldrich). Then 2 μg of total RNA was reverse transcribed to cDNA for RT-PCR using RevertAid First Strand cDNA Synthesis Kit (Thermo Scientific). Gene expression level of the mRNA of the related signaling molecules were cheeked more than Two times.

### Anti-obesity activity

Anti-obesity activities were determined using mouse embryo 3T3-L1 preadipocytes. First, cell viability was determined by MTT assay. Lipid droplets in 3T3-L1 cells were then differentiated under various doses of AnT Fr Adipocytes were extracted by Oil Red O staining and quantified with a spectrophotometer.

#### MTT assay

Toxicity of AnT Fr to 3T3-L1 Cells was determined by MTT assay. 3T3-L1 cells were cultured in 96-well plate at density of 1 × 10^4^ cells/well. These 96-well plates were kept in a humidified 5% CO2 incubator at 37°C for 24 hours. Cells were treated with different concentrations (5, 10,15, 20, 25, 30, 50, 100 and 200 μg/ml) of AnT Fr. After 24 hours of treatment with AnT Fr, MTT solution (0.5%) was added to each well of the plate containing cells and incubated for another 4 h. After aspiring the media, each well was added with dimethyl sulfoxide (DMSO) (Sigma) to dissolve formazan crystals. Absorbance was then read at wavelength of 540 nm on a microplate reader (Biochrom Ltd., UK). Cell viability (percentage) was calculated and compared to the control. Experiment was Two times repeated.

#### Oil Red O staining assay for anti-obesity determination

3T3-L1 cells were cultured in 6-well plates. After two days or when cell confluency reached 80%, media were replaced with differentiation induction media (10 μg/mL insulin, 1 mM dexamethasone, 0.5 mM Isobutyl-1-methylxanthine). Cells were kept in this state for 24 h to arrest cell division. After 2 days, culture media were replaced with maintenance media (DMEM media supplemented with 10 μg/insulin). These cells were then feed with maintenance media every 2 days up to 8 days. Samples of AnT Fr were added during the addition of induction media and maintenance media. GW9662 (10 μg/mL) was added as positive control as it is a potent antagonist of PPARγ inhibits adipogenesis. After 8 days of feeding phase, cells were washed with PBS and then fixed with formalin (10%) for 1 h. After cells were again washed with 60% iso-propanol, Oil Red O solution was added. Cells were then incubated at 37°C for 3 h. After cells were washed 4 times with distilled water and drying, they were washed with isopropanol to extract the staining dye. Effects of AnT Fr on lipids accumulation in cells were determined by photographing extracted Oil Red O solution of each group with a phase contrast microscope at 5 random sites. The absorbance of the extracted Oil Red O solution was then measured at wavelength of 520 nm using with a spectrophotometer. Experiments of the quantification of lipid droplets(triglycerides) accumulation in the 3T3-L1 deposits were performed 2 times.

#### Analysis of gene expression level

The inhibitory effect of AnT Fr on mRNA expression levels of adipogenesis and lipogenesis related gens and signal molecules was investigated by RT- PCR analysis using gene-specific primers. 3T3-L1 cells were cultured in DMEM media supplemented with 10% FBS in 6-well plate and incubated at 37°C in a CO_2_ incubator. These cells were then treated with different concentrations of AnT Fr and incubated in the CO_2_ incubator for 24 hours. Total RNA was isolated from these 3T3-L1 cells using TRI reagent (Sigma Aldrich) followed by DNase treatment. It was then quantified on a NanoDrop 2000 spectrophotometer. cDNA was then made from 500 ng of total RNA using Revert Aid Premium First Strand cDNA Synthesis Kit (Thermo Fisher Scientific). cDNA was quantified by checking absorption on a Nano Drop spectrophotometer. cDNA was then used for real-time qPCR with gene-specific primers. DNA bands were obtained on agarose gel after electrophoresis. RT-PCR analysis experiments were repeated more than 2 times.

### Western blotting

3T3-L1 adipocytes after differentiation were harvested at sub confluency, washed with PBS buffer and lysed with radioimmunoprecipitation assay buffer (Sigma-Aldrich, MO, USA) contained phenylmethylsulphonyl fluoride and protease inhibitor cocktail. Cell lysates were sonicated, centrifuged and supernatant was collected. protein concentration of the supernatant was measured by BCA protein Assay Reagent (Pierce, Rockford, IL, USA). 30 μg protein for each sample was separated by 10% sodium dodecyl sulfate (SDS) polyacrylamide electrophoresis (PAGE) and transferred onto nitrocellulose membranes (Bio-Rad, Hercules, CA). The membranes were then blocked in Tris-buffered saline (TBS)-Tween 20 solution containing 3% bovine serum albumin. After binding the membranes was then incubated overnight with AMPK, phospho-AMPKα (Cell Signaling Technology, Danvers, MA) and β-actin (Santa Cruz, CA) primary antibodies at 4°C. Secondary antibody was conjugated to horseradish peroxidase and enhanced chemiluminescence (Amersham Bioscience, UK) for visualizing protein bands. Experiments were repeated 2 times.

## Statistical analysis

Experiments were performed at least three times. Data are expressed as mean ± standard error of the mean (SEM). Statistical analysis was performed with analysis of variance (ANOVA) using SPSS followed by post-hoc comparison with Tukey’s test at α = 0.05. Statistical significance was considered at *p* < 0.05.

## Results

### Analysis and quantifications of anthocyanins rich fraction (AnT Fr)

The HPLC purified fraction of anthocyanins (AnT Fr) from ethanol leaf extract of *Cornus kusa* (ELECk) was subjected to GC-MS for identification and quantification of the present anthocyanins compounds (S1 Fig). 3 compounds, representing about 90.05% of the anthocyanins fraction AnT Fr of ELECk. The major compounds that were identified and quantified by GC–MS were cyanidin 3-glucoside, delphinidin 3-glucoside and pelargonidin 3-glucoside with the percent quantities as 27.23%, 36.54% and 26.32% respectively along with some other minor components presented in trace amounts.

### Anti-angiogenic activities

#### MTT assay

**Fig 1:**
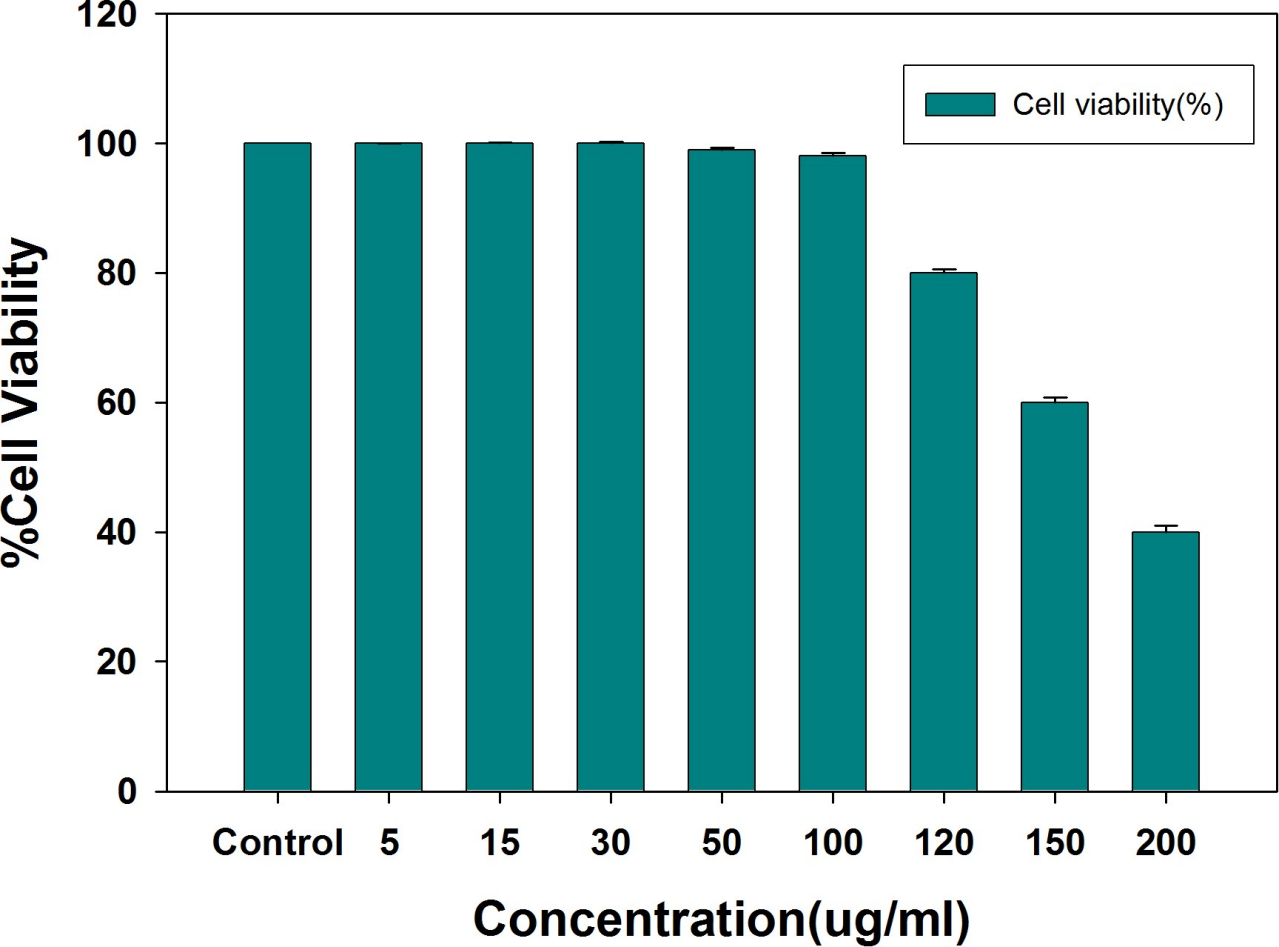
Determination of cell viability and toxicity of AnT Fr to HUVECs via MTT assay. HUVECs were cultured in EBM media in 96 well microtiter plate and after attachment different concentrations of AnT Fr were added to different wells contained same number of cells. Cells without treatment was taken as control. Cell viability was calculated for each group by checking the absorbance of the wells after addition of MTT reagent. Safe and toxic concentrations were determined. Data are expressed as mean ± SEM of three different experiments. (* p < 0.05 and ** p < 0.01 as compared to control).

Anti-angiogenic potential of AnT Fr was determined using HUVECs. First, cell viability was determined by MTT assay and safe doses were determined. HUVCEs were allowed to proliferate in the presence of different concentrations of AnT Fr in 96-well microtiter plate with equal number of cells. Wells of the plate containing HUVCEs without treatment with AnT Fr were used as negative control. EGCG (Epigallocatechin gallate) was used as positive control (50 μg/ml). Toxicity of EGCG to HUVECs was first determined by MTT assay (S2 Fig). MTT assay results shown that AnT Fr do not pose any toxicity to HUVECs up to concentration of 100 μg/ml (Fig 1).

Only safe (nontoxic) doses of AnT Fr were selected for further experiments to determine its anti-angiogenesis activities. Effect of AnT Fr of ELECk on human erythrocytes was evaluated in order to determine its hemolysis effects. The used dosses of AnT Fr in experiments did not show hemolytic effects after incubation for 240 minutes indicating the safety of the anthocyanins rich fraction (AnT) to normal cells (S3 Fig).

#### Anti-angiogenic/tube formation-inhibiting activities

**Fig 2:**
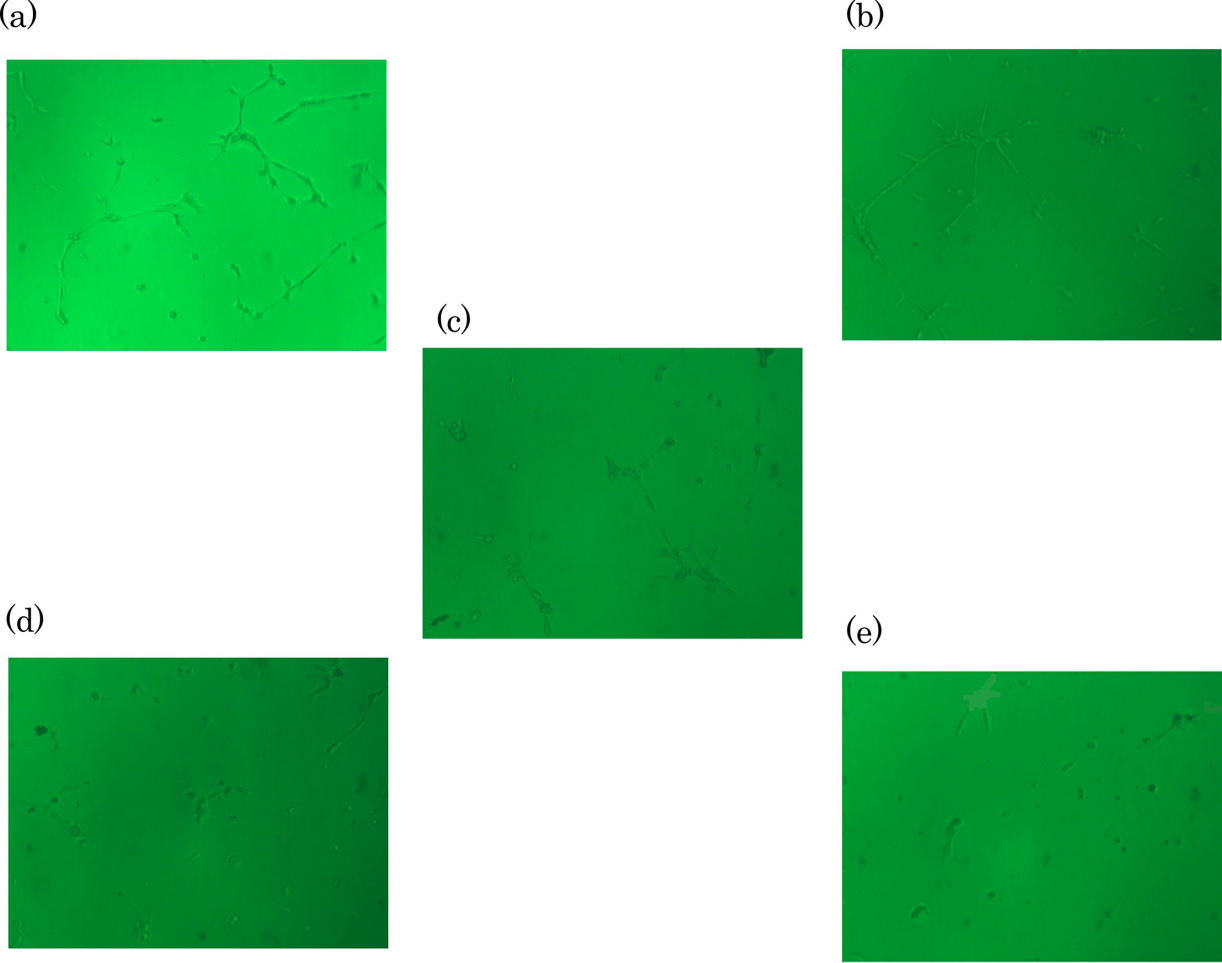
Inhibitory effects of AnT Fr on tubes formation by HUVECS. Cells were allowed to proliferate in EBM2 media in the presence and absence of various concentrations i.e. 5, 30, 60 and 100 μg/mL (nontoxic range) of AnT Fr and tubes formation by HUVECs in Matrigel was observed with the help of a phase contrast inverted microscope (Nikon, Tokyo, Japan) and photographed with Scion Image software (NIH, ML, USA) at 5 random sites. **A.** Tubes formation by HUVECs in control (without AnT Fr treatment) group. **B.** Tubes formation by HUVECs under treatment with 5 μg/mL AnT Fr. **C.** Tubes formation by HUVECs under treatment with 30 μg/mL ELECk. **D.** Tubes formation by HUVECs under treatment with 60 μg/mL AnT Fr. **E.** Tubes formation by HUVECs under treatment with 100 μg/mL AnT Fr.

Antiangiogenic effect of AnT Fr was explored by determining its inhibitory effect on the formation of tubular structure by HUVECs in Matrigel. HUVECs were grown in 96-well microtiter plate. After reaching confluency, cells were treated with various concentrations of AnT Fr. They were allowed to proliferate and make tubular structures in Matrigel under the influence of AnT Fr. HUVECs of each group proliferated under AnT Fr were photographed at 5 random sites with a phase contrast inverted microscope. HUVECs in the negative and positive control group was also photographed randomly (S4 Fig). The effect of different concentrations of AnT Fr on HUVECs proliferation in Matrigel was determined (Fig 2).

**Fig 3:**
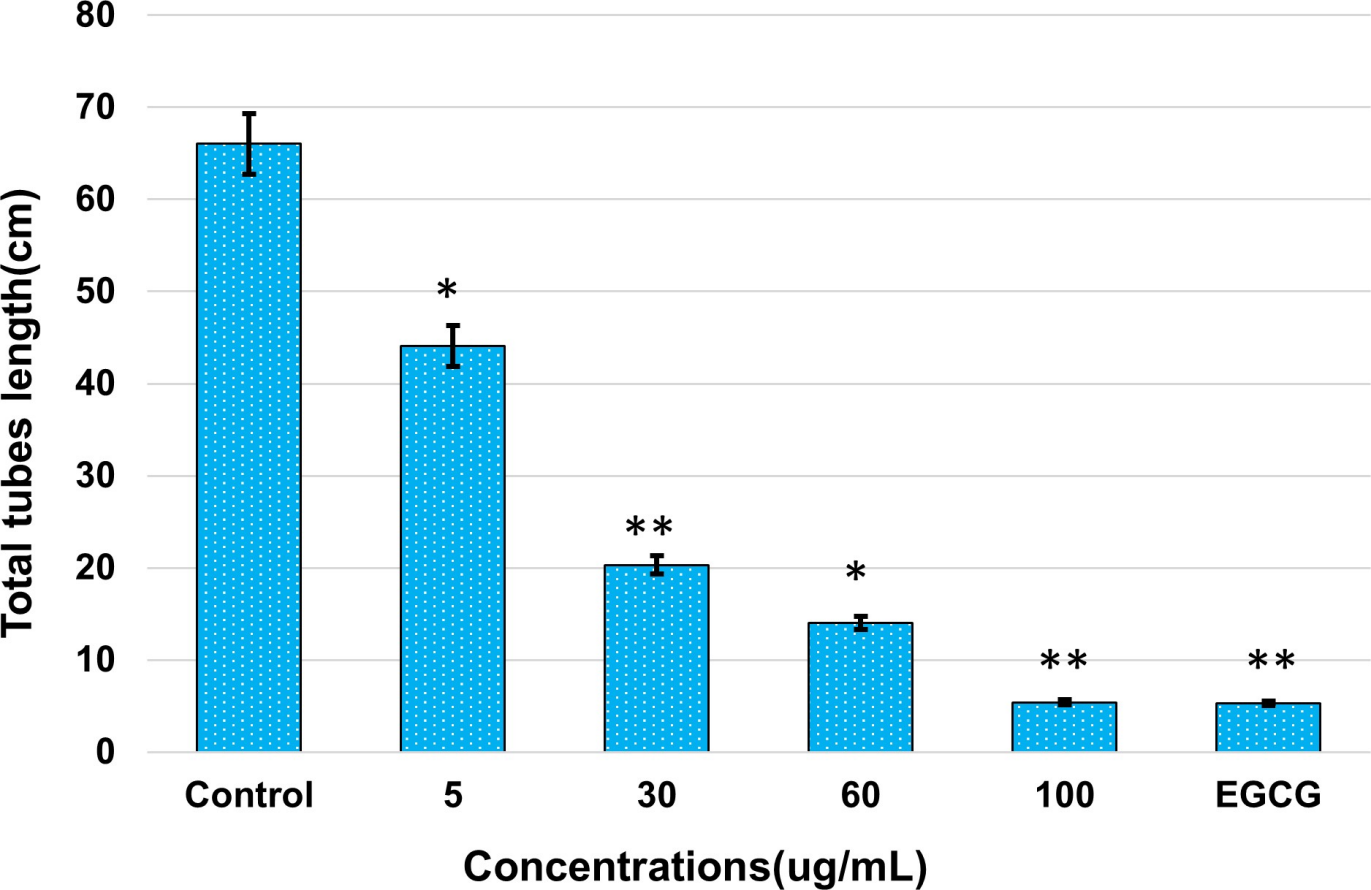
Inhibitory effects of AnT Fr on HUVECS proliferation i.e. tubes formation. Cells were allowed to proliferate in EBM2 media in the presence and absence of various concentrations i.e. 5, 30, 60 and 100 μg/mL (nontoxic range) of AnT Fr and tubes formation by HUVECs in Matrigel was observed with the help of a phase contrast inverted microscope (Nikon, Tokyo, Japan) and photographed with Scion Image software (NIH, ML, USA) at 5 random sites. Total tubes length was then calculated for each group. Experiments were performed in triplicate. Data are the mean values ± SEM. (* p < 0.05 and ** p < 0.01 as compared to control).

Total tube length was calculated for each concentration and compared with the control. Results showed that AnT Fr significantly inhibited HUVECs proliferation in a dose-dependent manner. Total tube length was found to be decreased with increasing concentrations of AnT Fr (Fig 3). Total tube length was the highest in the control. It was the lowest in the group treated with the highest concentration (i.e., 100 μg/ml) of ELECk, indicating that suppression of angiogenesis in HUVECs by ELECk was in a dose-dependent manner.

#### RT-PCR analysis

The mechanisms of the antiangiogenic activities of AnT Fr in HUVECs was analyzed by RT-PCR. Total RNA was extracted from the treated and control cells and reverse transcribed to cDNA. The resulted cDNA was subjected to Real time PCR using gen specific primers (Table 1).

**Table 1:** Gene-specific primers along with their product size and gene bank accession number used for PCR for antiangiogenic activities determination.

Gene	Gene Bank Accession No.	Primer sequence	Size (bp)
VEGFR-2	NM_002253.2	F -AGG TTG CGT GTT CTT CGA GT R -CCC AAA GTG CTG GGT TTT TA	934
PI3K	NM_006218.2	F -CGT GTG CCA TTT GTT TTG AC R -TCA AAC CCT GTT TGC GTT TAC	536
NF-kB	NM_001145138.1	F -TGG TCA GCT CCC TTC TCT GT R -GCC AGC TTG GCA ACA GAT	521
AKT-1	NM_005163.2	F -CCG ATT CAC GTA GGG AAA TG R -AGC GTC GAA AAG GTC AAG TG	529
β-Catenin	NM_001098209.1	F -GGT GGG CTG GTA TCT CAG AA R -GGC AAC TGG TAA ACT GTC CAA	629
β-actin	NM_001101.3	F -CTC CTG AGC GCA AGT ACT CC R -ACA TCT CAA GTT GGG GGA CA	632

F and R indicates Forward and reverse sequences respectively

**Fig 4:**
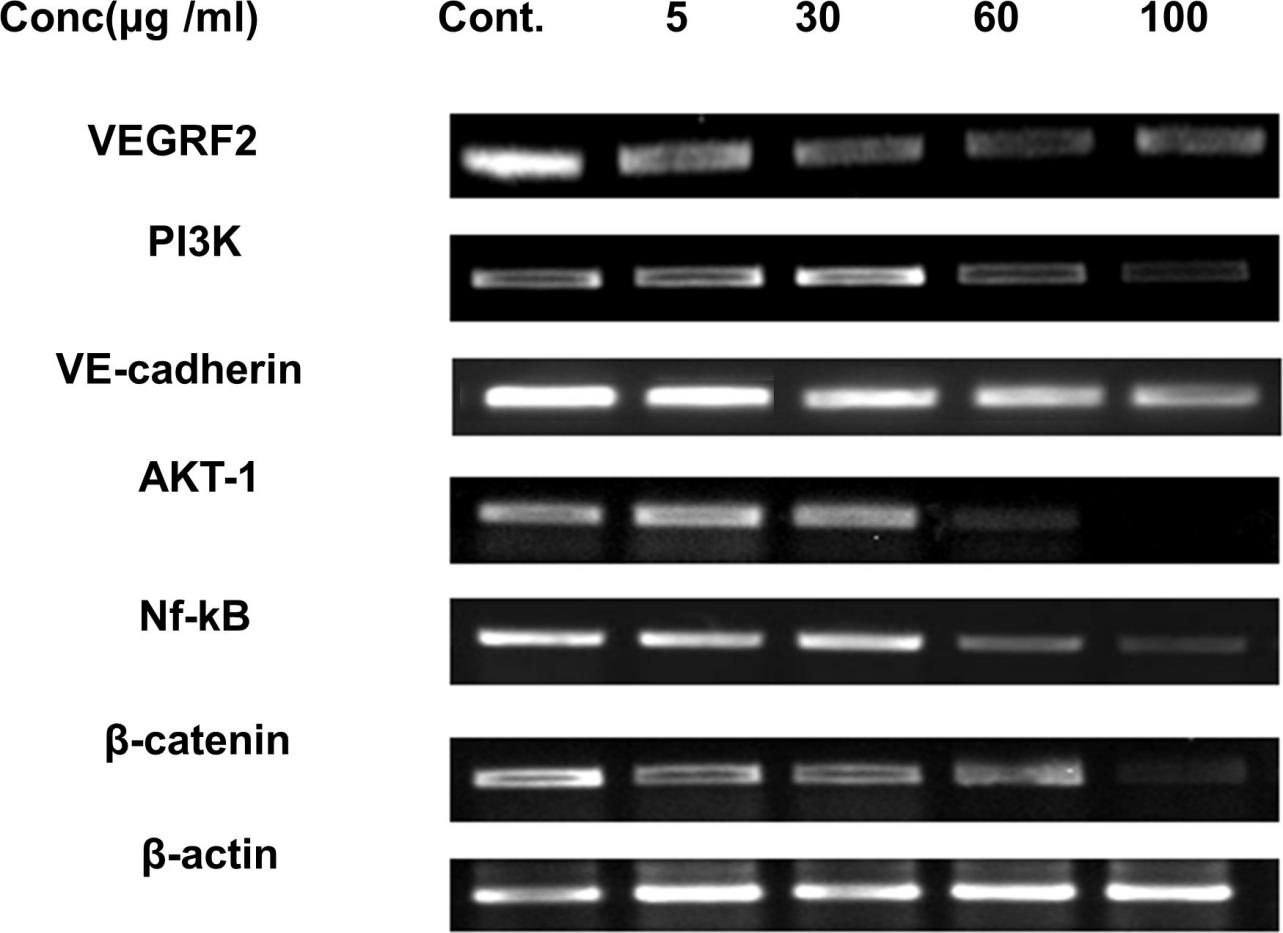
Gene expression level of various angiogenesis promoting signaling molecules in HUVECs treated with various dosses of AnT Fr. HUVECs were proliferated under different concentration of AnT Fr and total RNA was extracted, and reverse transcribed to cDNA which was then amplified by real time quantitative PCR using gene specific primers.

The product was then visualized by obtaining bands by Gel doc after gel electrophoresis. AnT Fr significantly decreased expression levels of angiogenic signal molecules such as VEGFR2, PI3k, Akt, and Nf-Kb in a dose-dependent manner (Fig 4). The highest safe dose of AnT Fr (i.e., 100 μg/ml) showed the highest inhibition on angiogenesis process in HUVECs by suppressing signaling proteins and transcriptional factors of angiogenesis.

### Anti-obesity activity

Anti-obesity effect of AnT Fr was determined by antihyperlipidemic activities via antiadipogenic and anti-lipogenic potential of AnT Fr.

#### MTT assay

**Fig 5:**
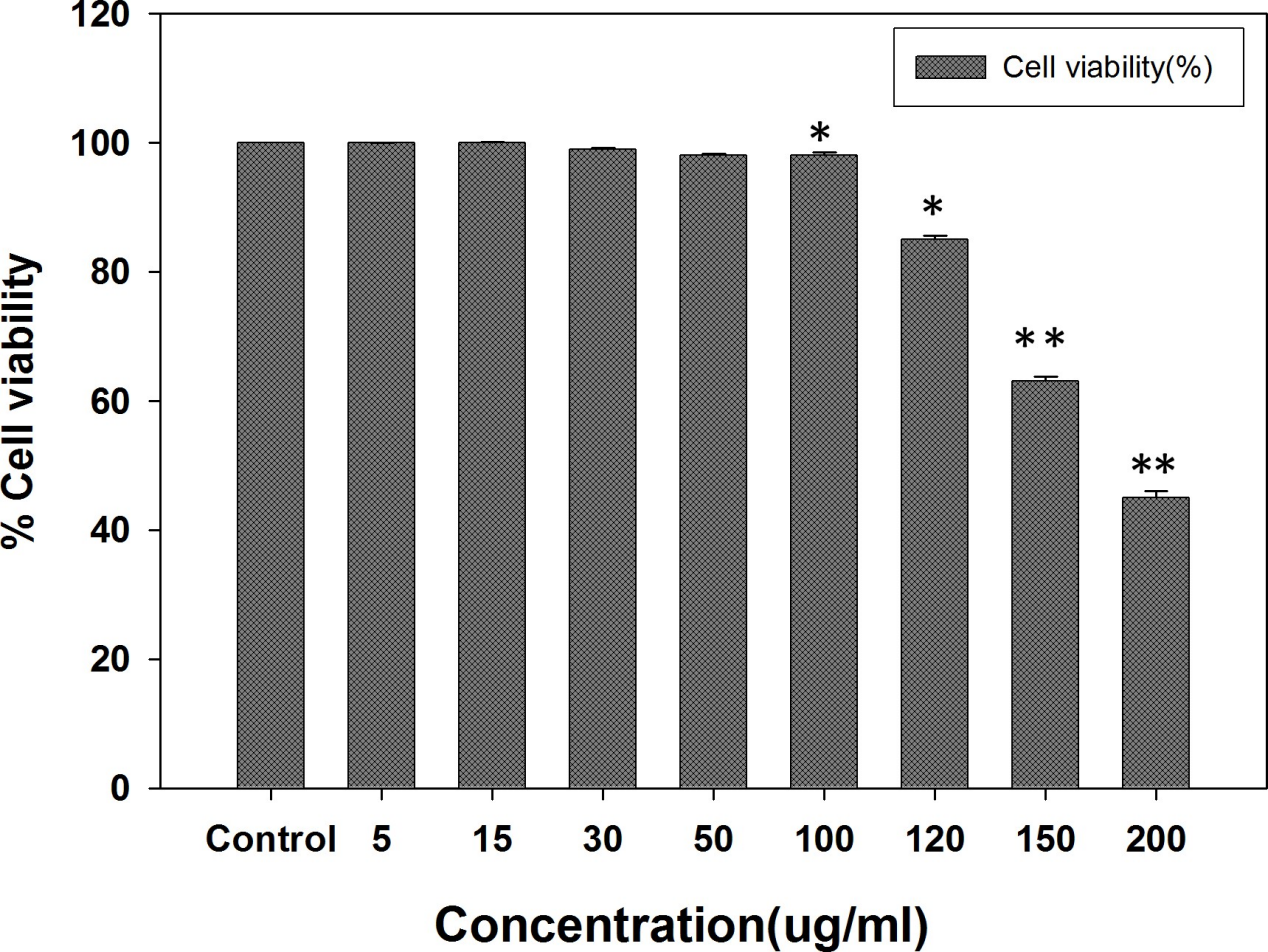
Viability of 3T3-L1 adipocytes under various concentrations of AnT Fr determined by MTT assay. 3T3-L cells were cultured in DMEM media in 96 well microtiter plate and after attachment different concentrations of AnT Fr were added to different wells contained same number of cells. Cells without treatment was taken as control. Cell viability was calculated for each group by checking the absorbance of the dissolved formazan crystals in DMSO after addition of MTT reagent. Safe and toxic concentrations were determined. Data are expressed as mean ± SEM of three different experiments. (* p < 0.05 and ** p < 0.01 as compared to control).

For antihyperlipidemic activities, first the cell viability assay was performed by MTT using 3T3-L1 cell line for determination of safe and toxic concentrations level. AnT Fr did not show any toxicity to 3T3 -L1 cells at concentration up to 100 μg/ml. At concentration higher than 100 μg/ml, AnT Fr deceased viability of 3T3 -L1 cells (Fig 5). Thus, safe doses of AnT Fr were determined as up to 100 μg/ml for further experiments.

Toxicity of the positive controls EGCG and GW9662 to 3T3-L1 cells was also determined by MTT assay before using for further experiments (S5 Fig).

#### Oil Red O staining assay

**Fig 6:**
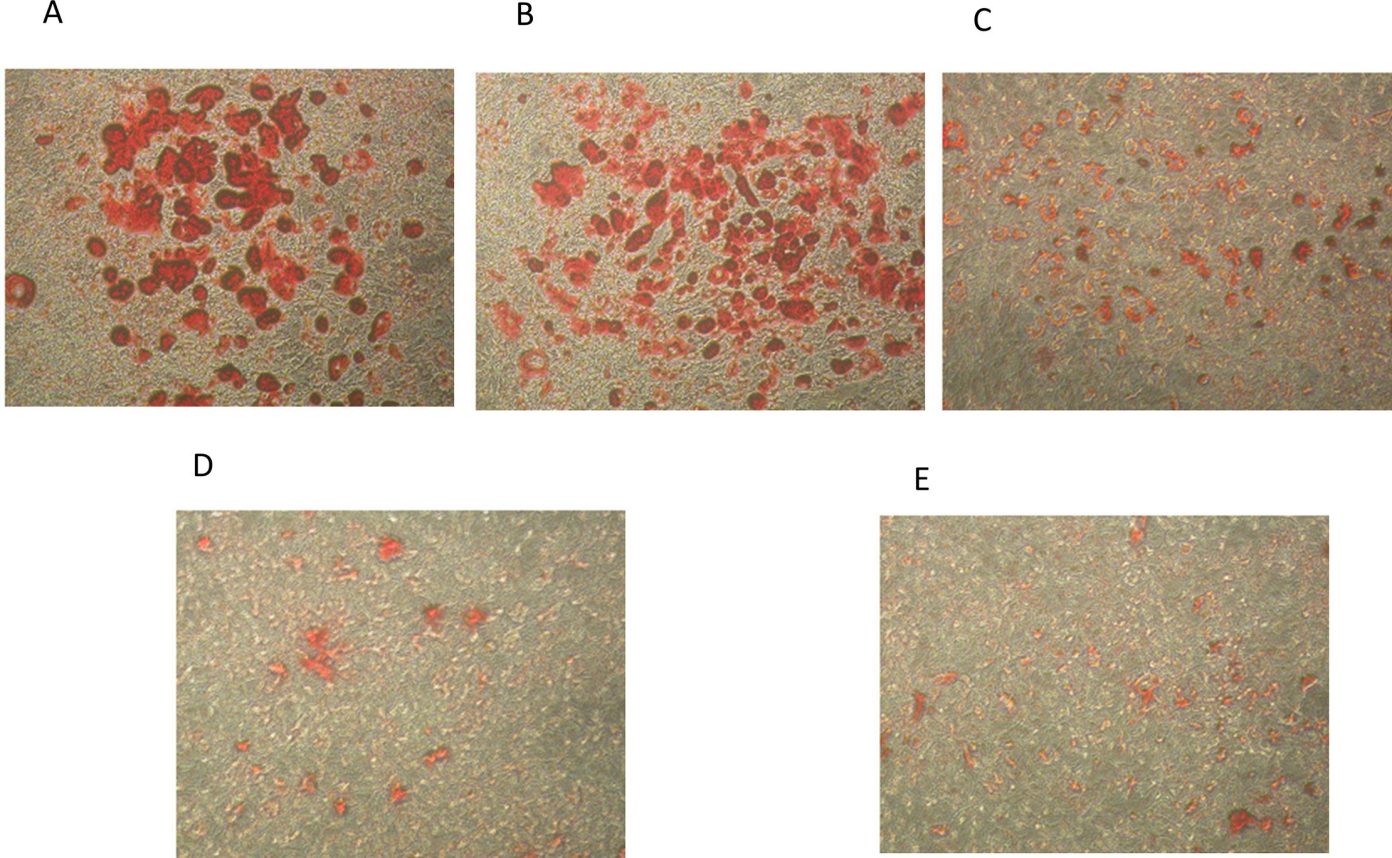
Inhibition of fats accumulation in 3T3-L1 cells via suppression of adipogenesis and lipogenesis by AnT Fr. 3T3-L1 pre-mature cells were allowed to proliferate in DMEM media in the presence (various concentrations) and absence (control) of ELECk. the lipid droplets accumulated in mature 3T3-L1 cell were observed with the help of a phase contrast inverted microscope (Nikon, Tokyo, Japan) and photographed with Scion Image software (NIH, ML, USA) at 5 random sites. **A.** Fats accumulated in differentiated 3T3-L1 cells of control (untreated) group. **B.** Fats accumulated in differentiated 3T3-L1 cells of group treated with 5 μg/mL of AnT Fr. **C** Fats accumulated in differentiated 3T3-L1 cells of group treated with 30 μg/mL of AnT Fr. **D.** Fats accumulated in differentiated 3T3-L1 cells of group treated with 60 μg/mL of AnT Fr **E.** Fats accumulated in differentiated 3T3-L1 cells of group treated with 100 μg/mL of AnT Fr.

Pre-mature 3T3-L1 cells were differentiated in the presence of various concentrations (nontoxic level) of AnT Fr. Lipid droplets accumulated in the cells were extracted with Oil Red O staining solution and was quantified with a spectrophotometer by checking the absorbance of the extracted Oil Red O staining. Photographs of lipid droplets in Oil Red O staining solution showed that lipid accumulation was significantly reduced with increasing concentrations of AnT Fr (Fig 6).

**Fig 7:**
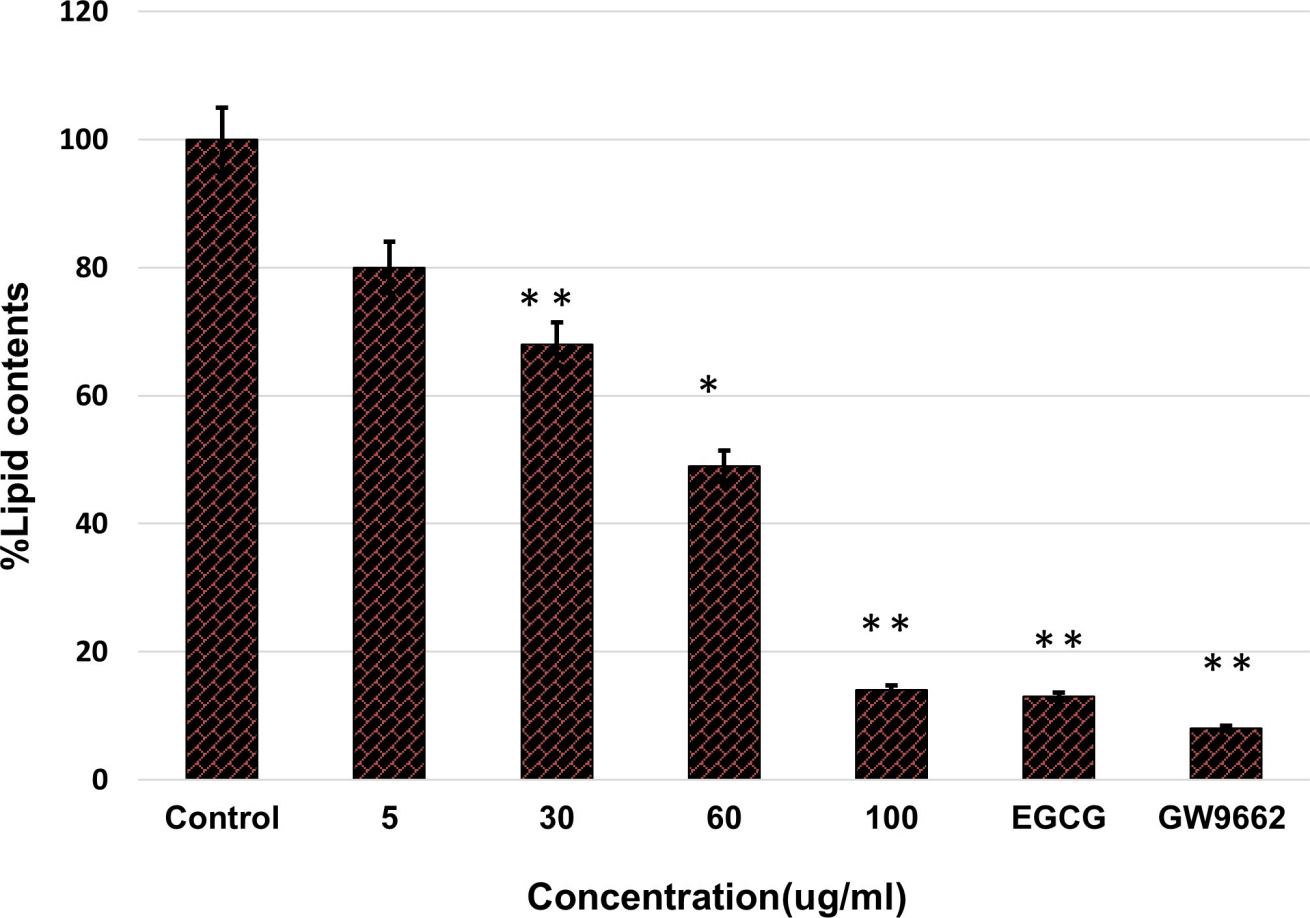
Inhibition of fats accumulation in 3T3-L1 via suppression of adipogenesis and lipogenesis by AnT Fr determined by Oil O Red solvent extraction. 3T3-L1 pre-mature cells were allowed to proliferate in DMEM media in control and ELECk treated groups. The lipid droplets accumulated in mature differentiated 3T3-L1 cell were extracted with Oil O Red solvent and quantified by checking absorbance with the help of a micro titer plate reader spectrophotometer. Data are expressed as mean ± SEM of three different experiments. (* p < 0.05 and ** p < 0.01 as compared to control).

AnT Fr inhibited lipid accumulation in differentiated 3T3-L1 adipocytes in a dose-dependent manner as compared to controls. The highest concentration of AnT Fr supressed lipid accumulation in 3T3-L1 cells nearly the same as GW9662, the positive control (Fig 7)

#### RT-PCR

The mechanisms of the antihyperlipidimic activities of AnT Fr on 3T3-L1 proliferation and lipid accumulation was analyzed by RT-PCR. Total RNA was isolated from 3T3-L1 cells proliferated under the presence of various concentrations of AnT Fr. RNA was reverse transcribed to cDNA by reverse polymerase chain reaction and then converted to DNA by real time qPCR using gen specific primers (Table 2).

**Table 2:** Gene-specific primers used for adipogenesis and lipogenesis related PCR along with the product size and gene bank accession number details.

Gene	Gene bank accession no.	Primer sequence	Size (bp)
SREBP	NM_033218.1	F -TTG CAC CAG AGA GCA TTT TG-3’ R -GAA AAT GAG AGG CTG GTT GC-3’	593
C/EBPα	NM_007678.3	F -TTA CAA CAG GCC AGG TTT CC-3’ R -CCA CAG GGG TGT GTG TAT GA-3’	629
PPARγ	NM_001127330.1	F -CTG GCC TCC CTG ATG AAT AA-3’ R -GGG TGA AGG CTC ATG TCT GT-3’	393
aP2	NM_024406.2	F -CAG CCT TTC TCA CCT GGA AG-3’ R -TCG ACT TTC CAT CCC ACT TC-3’	352
FAS	NM_007988.3	F -AAA GGA CCT GCC CAA TCT CT-3’ R -TGA TCA AAC TCA GGC TGC AC-3’	245
LPL	NM_008509.2	F -AAG CCC CAC AAG TGT AGT CG-3’ R -CGG ACA CAA AGT TAG CAC CA-3’	402
Actin	NM_007393.3	F-GTT GGT TGG AGC AAA CAT CC-3’ R-GAG GGT GAG GGA CTT CCT GT-3’	151

F and R indicates Forward and reverse sequences respectively

**Fig 8:**
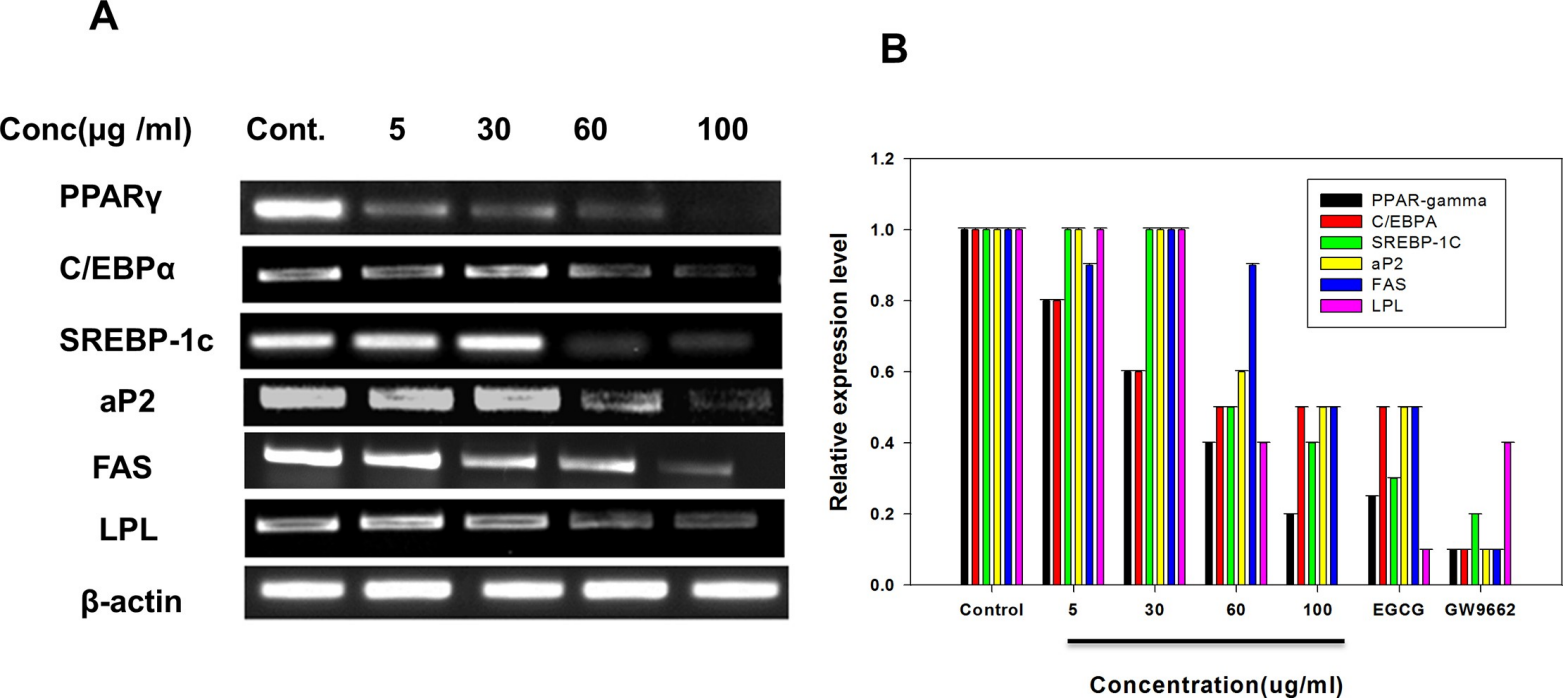
Effect of AnT Fr on mRNA levels of adipogenesis and lipogenesis related genes and signaling molecules in 3T3- L1 adipocytes. 3T3-L1 preadipocytes were differentiated to adipocytes in presence of various concentrations of AnT Fr. Total RNA was extracted from 3T3-L1 cells of each group and converted to cDNA and then DNA by RT-PCR. **A.** mRNA expression levels of adipogenesis and lipogenesis related gens and signaling proteins visualized by gel electrophoresis. **B.** Expression levels of relative mRNA of adipogenesis and lipogenesis and signaling proteins, measured using a quantitative real-time RT–PCR. mRNA amount of each group was normalized to that of b-actin and expressed relative to control group. Experiments were performed in triplicate and data are expressed as mean ± SEM (* p < 0.05 and ** p < 0.01 as compared to control).

The product was then visualized by obtaining bands by Gel doc after gel electrophoresis. RT-PCR rresults showed that AnT Fr significantly inhibited expression levels of adipogenesis and lipogenesis related genes and signal molecules such as PPARγ, C/EBPα, aP2, and SREBP-1c in a dose-dependent manner (Fig 8).

### Effect of ELECk on activation of p-AMPK

**Fig 9:**
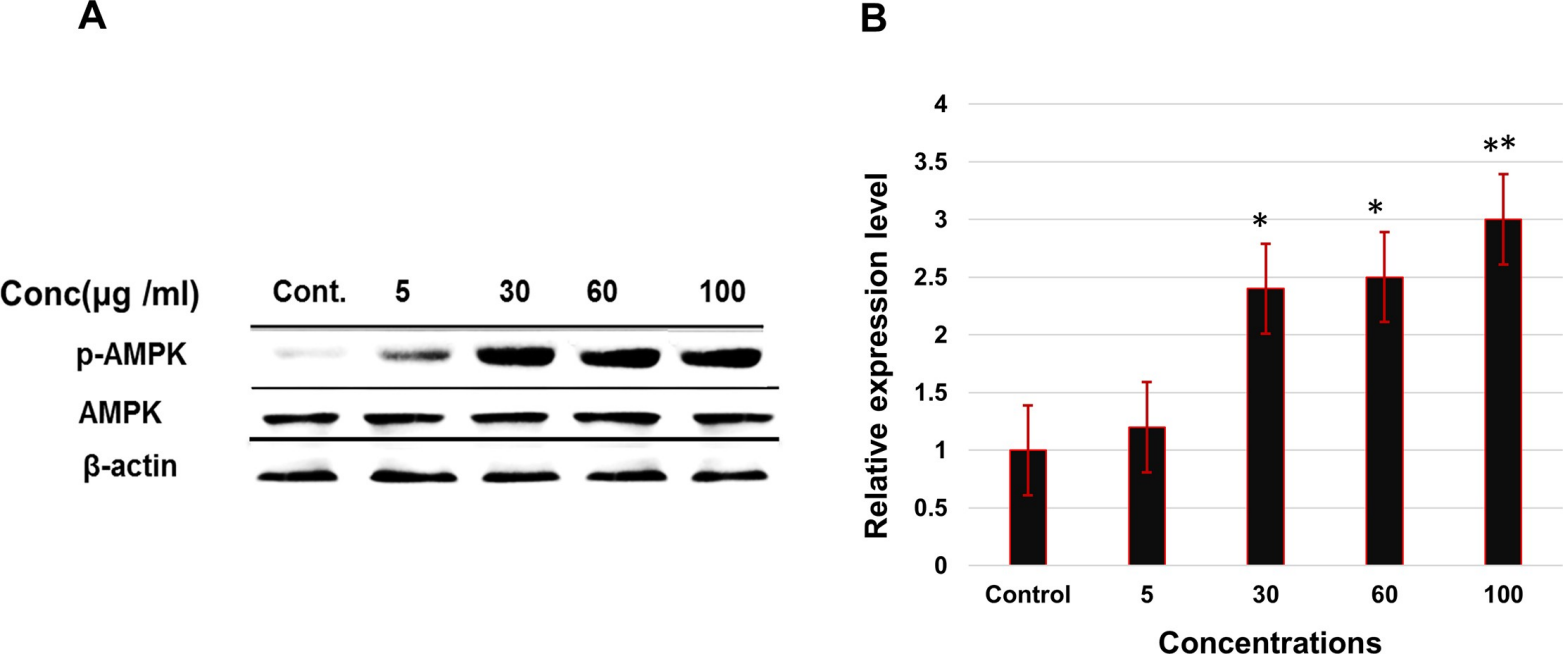
Effect of AnT Fr on p-AMPK activation determined by western blotting. 3T3-L1 preadipocytes were differentiated to adipocytes in presence of various concentrations of AnT Fr. Protein level was expressed as the fold relatively after normalized by b-actin level. **A.** Proteins bands visualized after western blotting for AMPK, p-AMPKα and β-actin. **B.** Relative expression levels of phospho-AMPKα proteins determined by western blotting. Experiments were performed in triplicate and data are expressed as mean ± SEM (* p < 0.05 and ** p < 0.01 as compared to control).

Activation of p-AMPK by AnT Fr was determined by western blot analysis. 3T3-L1 adipocytes were differentiated under treatment with various concentrations of AnT Fr. Proteins were extracted with radioimmunoprecipitation assay buffer and concentration was measured by BCA protein Assay Reagent (Pierce, Rockford, IL, USA). Protein separated by SDS-PAGE were transferred onto nitrocellulose membranes was then incubated overnight with AMPK, phospho-AMPKα (Cell Signaling Technology, Danvers, MA) and β-actin (Santa Cruz, CA) primary antibodies at 4°C. Secondary antibody was conjugated to horseradish peroxidase and enhanced chemiluminescence Amersham Bioscience, UK for visualizing protein bands. The result shown that AnT Fr has effect on activation of p-APMK in a dose dependent manner. The expression level of the p-APMK shows enhancement with increasing concentration of AnT Fr as compared to AMPK and beta actin level which was taken as control (Fig 9).

## Discussion

Obesity is a multifactorial metabolic disorder and a risk factor for diabetes, cancer, cardiovascular diseases, and inflammatory diseases [22,23]. Lipid accumulation increases the size of cells, resulting in expansion of adipose tissues. Lipid synthesis occurs as a result of adipogenesis and lipogenesis regulated by a number of signaling pathways. These pathways involve many signal molecules and transcription factors such as peroxisome proliferator-activated receptor γ (PPARγ) and CCAAT/enhancer-binding protein α (C/EBPα) that play a key role in adipocyte differentiation. PPARγ regulates the expression of adipogenesis promoting genes [10,11]. PPARγ assists C/EBPα expression and induces adipocyte differentiation by activating aP2 and LPL [12] (Song Y *et al*., 2013). SREBP-1c is crucial for lipogeneses promotion via activating transcriptional factors SCD-1, FAS, ACC, and PPARγ [24–26]. AMPK is a key factor that regulates glucose and lipids metabolism. Phosphorylated AMPK inactivates key enzymes involved in fatty acid and sterol synthesis such as fatty acid synthetase (FAS) and acetyl-CoA carboxylase (ACC-1). It enhances fatty acid oxidation [27]. AMPK controls the expression of PPARγ, C/EBPα, and SREBP [28, 29]. AMPK activation and overexpression will downregulate adipogenesis and lipogenesis.

Natural products are significant therapeutic compounds with multiple molecular targets. They are preferred alternatives to synthetic chemical compounds [30,31]. Various natural products have been used to treat different diseases by modulating, enhancing, or suppressing transcriptional factors and signaling proteins. Anti-obesity activities of natural compounds are mediated by regulating various pathways (including lipid absorption, energy intake and expenditure), increasing lipolysis, and decreasing lipogenesis, differentiation, and proliferation of preadipocytes [30].

The genus *Cornus* commony known as dogwood consists of about 55 species and several species have been reported for analgesic, anti-malarial, diuretic anti-bacterial, anti-allergic anti-histamine and anti-microbial activities and are used in Chinese herbal medicine [32]. Cronus spp. Contained Anthocyanin, the bioactive flavonoids with therapeutic effects which have been reported to possess antioxidant, anti-inflammatory, anticancer and anti-diabetic activities [33, 34]. Anthocyanin possess antihyperlipidemic effects and reduces serum cholesterol level [35]. It has been studied that anthocyanin effectively reduce fats level in C57BL/6J mice fed with high lipids diet [36]. Peng et al., [37] reported significant reduction of accumulated body fats by anthocyanins rich mulberry water extracts. Anthocyanins modulate obesity and reduce inflammation by regulating MAPK and NF-κB signaling pathways [38]. Elena et al., [39] reported anti-obesity activities of anthocyanin supplementation. In our study we investigated the inhibitory effects of *Cornus kusa* extracted anthocyanins on angiogenesis, adipogenesis and lipogenesis. In this study, we experimentally proved that AnT Fr could inhibit lipid accumulation during differentiation of 3T3-L1 adipocytes without posing toxicity to normal cells. Its action of mechanisms was elucidated by measuring expression levels of adipogenesis and lipogenesis related genes and proteins in 3T3-L1 adipocytes by RT-PCR and western blot analysis. AnT Fr significantly suppressed expression levels of adipogenesis/lipogenesis-associated genes and proteins in 3T3-L1adipocytes. It also upregulated protein expression levels of p-AMPK. Treatment with AnT Fr decreased expression levels of signaling proteins PPARγ and C/EBPα and adipogenesis related gens aP2 and LPL in a dose-dependent manner. Similarly, mRNA expression levels of lipogenesis related genes (such as SCD-1, FAS, and ACC) and protein expression levels of SREBP-1c and FAS were decreased in 3T3-L1 cells after treatment with AnT Fr. Angiogenesis assists obesity by feeding adipose tissues. Inhibition or suppression of angiogenesis contributes to inhibition of fat accumulation in adipocytes, resulting in downregulation of obesity. We investigated the antiangiogenic potential of AnT Fr in HUVECs determined by the inhibition of tubular structures (i.e., vessels formation by HUVECs) in Matrigel. Our results showed that AnT Fr inhibited tube formation of HUVECs in a dose-dependent manner. RT-PCR analysis revealed that AnT Fr downregulated angiogenesis by suppressing expression levels of angiogenesis promoting signaling proteins.

The anti-obesity and anti-angiogenic activities of AnT Fr might be due to the presence of anthocyanins i.e. delphinidin 3-glucoside, cyanidin 3-glucoside, pelargonidin 3-glucoside.

## Supporting information

S1 Fig HPLC and GC-MS analysis for isolation and molecular characterization of AnT Fr of ELECk. (DOCX)

S2 Fig Determination of EGCG toxicity to HUVECs cells by MTT assay. (DOCX)

S3 Fig Hemolysis effect of AnT Fr of ELECk on human erythrocytes. (DOCX)

S4 Fig Effects of the positive control i.e. EGCG and GW9662 on HUVECs tubes formation and lipids accumulation in 3T3-L1 adipocytes. (DOCX)

S5 Fig Determination of EGCG and GW9662 toxicity to 3T3-L1 cells by MTT assay. (DOCX)

S6 Fig Graphical abstract. (TIF)

S1 File Isolation of anthocyanins rich fraction (AnT Fr) from ELECk by HPLC and molecular characterization by GC-MS. (DOCX)
