# Supplementary material for: Anthocyanins from Cornus kousa ethanolic extract attenuate obesity in association with anti-angiogenic activities in 3T3-L1 cells by down-regulating adipogeneses and lipogenesis
Source: PLoS One. 2018 Dec 6;13(12):e0208556. doi: 10.1371/journal.pone.0208556 (PMC6283641; doi:10.1371/journal.pone.0208556)
Supplement: S2 Fig — (DOCX) [file pone.0208556.s002.docx]

**S2 Fig. Determination of EGCG toxicity to HUVECs cells by MTT assay. Data are mean values (n=3) ±SEM. Data are statistically significant at P<0.005**
