# Supplementary material for: Anthocyanins from Cornus kousa ethanolic extract attenuate obesity in association with anti-angiogenic activities in 3T3-L1 cells by down-regulating adipogeneses and lipogenesis
Source: PLoS One. 2018 Dec 6;13(12):e0208556. doi: 10.1371/journal.pone.0208556 (PMC6283641; doi:10.1371/journal.pone.0208556)
Supplement: S3 Fig — (DOCX) [file pone.0208556.s003.docx]

**S3 Fig. Hemolysis effect of AnT Fr of ELECk on human erythrocytes.** **The used dosses of AnT Fr in experiments did not show hemolytic effects after incubation for 240 minutes indicating the safety of the anthocyanins rich fraction (AnT) to normal cells. Date are expressed as mean values(n=3) ± SEM. Data are statistically significant at p<0.005**
