# Supplementary material for: Anthocyanins from Cornus kousa ethanolic extract attenuate obesity in association with anti-angiogenic activities in 3T3-L1 cells by down-regulating adipogeneses and lipogenesis
Source: PLoS One. 2018 Dec 6;13(12):e0208556. doi: 10.1371/journal.pone.0208556 (PMC6283641; doi:10.1371/journal.pone.0208556)

**S4 Fig. Tubes formation by HUVECs in positive control group (EGCG 50 μg/ml) (a). Lipids accumulation after Oil Red O staining in positive control group i.e. EGCG (50 μg/ml) (b) and** **GW9662 (10 μg/ml) (c). Photo graphs were taken by phase contrast inverted microscope connected to a digital camera.**


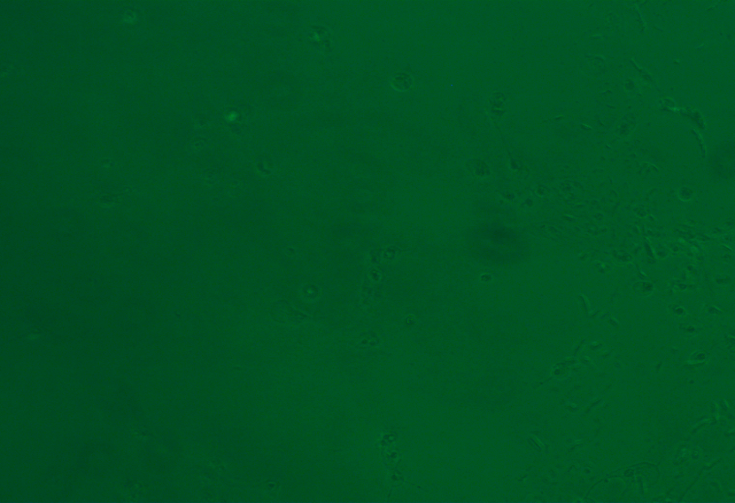

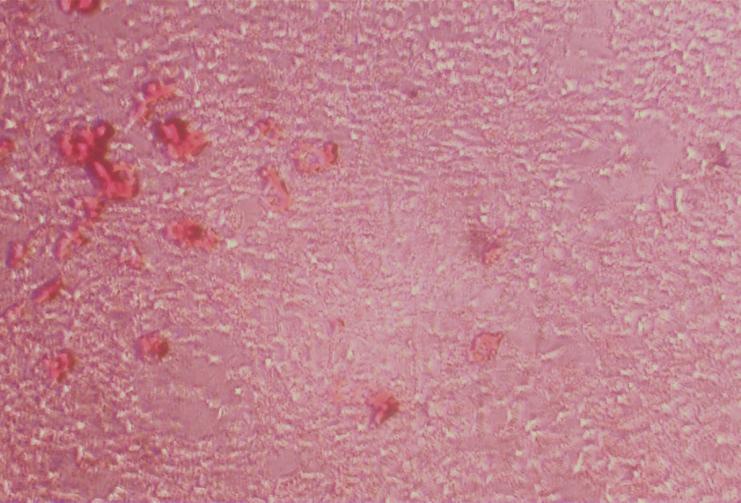
**a b**

**c**


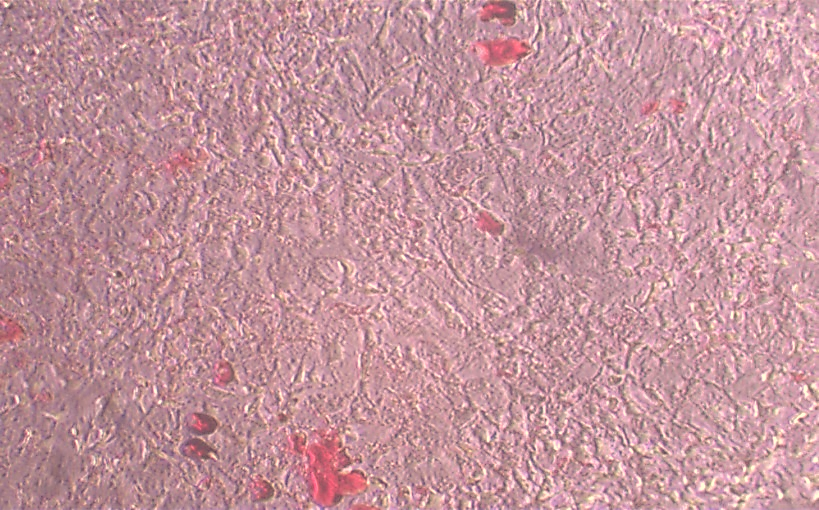

Supplement: S4 Fig — (DOCX) [file pone.0208556.s004.docx]
