# Supplementary material for: Anthocyanins from Cornus kousa ethanolic extract attenuate obesity in association with anti-angiogenic activities in 3T3-L1 cells by down-regulating adipogeneses and lipogenesis
Source: PLoS One. 2018 Dec 6;13(12):e0208556. doi: 10.1371/journal.pone.0208556 (PMC6283641; doi:10.1371/journal.pone.0208556)
Supplement: S5 Fig — (DOCX) [file pone.0208556.s005.docx]

**S5 Fig. Determination of EGCG and GW9662 toxicity to 3T3-L1 cells by MTT assay respectively. Data are mean values (n=3) ±SEM. Data are statistically significant at P<0.005**
