# Supplementary figures and images for: Anthocyanins from Cornus kousa ethanolic extract attenuate obesity in association with anti-angiogenic activities in 3T3-L1 cells by down-regulating adipogeneses and lipogenesis
Source: PLoS One. 2018 Dec 6;13(12):e0208556. doi: 10.1371/journal.pone.0208556 (PMC6283641; doi:10.1371/journal.pone.0208556)

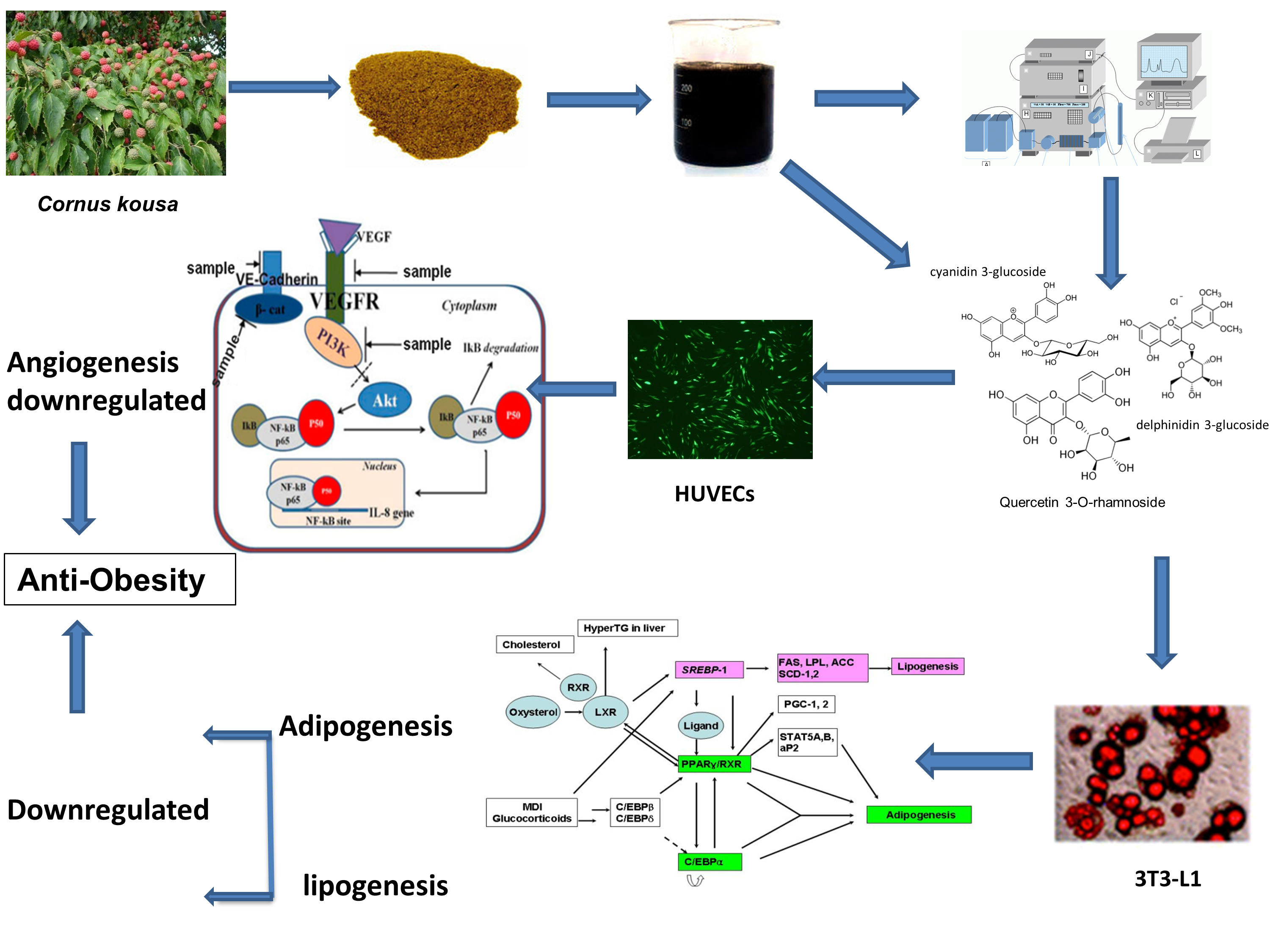

Supplement: S6 Fig — (TIF) [file pone.0208556.s006.tif]
