# Supplementary material for: Anthocyanins from Cornus kousa ethanolic extract attenuate obesity in association with anti-angiogenic activities in 3T3-L1 cells by down-regulating adipogeneses and lipogenesis
Source: PLoS One. 2018 Dec 6;13(12):e0208556. doi: 10.1371/journal.pone.0208556 (PMC6283641; doi:10.1371/journal.pone.0208556)
Supplement: S1 File — (DOCX) [file pone.0208556.s007.docx]

**S1 File. Isolation of anthocyanins rich fraction (AnT Fr)** **from ELECk by HPLC and molecular characterization by GC-MS**

**HPLC**

The Anthocyanins rich fraction (AnT Fr) used in this study was isolated from ethanolic leaf extract of *Cornus kousa* (ELECk) by high-pressure liquid chromatography (HPLC). The system (Shimdzu HPLC) equipped with

- Pump: Shimadzu LC-20AD

- Detector: Photodiode array detector, Shimadzu SPD-M20A

- Degasser: Shimadzu DUG-20A3

- Oven: Shimadzu CTO-20A

- Autosampler: Shimadzu SIL-20A

- System controller: CBM-20A

- Software: Shimadzu Solution (ver.1.22sp)

**HPLC condition**

Column: Luna C18(2) (150 x 3.0mm, 3μm)

Mobile Phase: A: 0.1% Formic acid in acetonitrile

B: 0.1% formic acid in water

Gradient: From A/B (33:67) to (18:65.5) in 90 min to (100:0) in 1min.

Hold A/B (100:0) for 8min and A/B (33:67) for 20min

Flow Rate: 0.34mL/min

Temperature: 35℃

Detector: UV at 205nm

**GC-MS analysis**

3 compounds, representing about 90.05% of the anthocyanins fraction AnT Fr of ELECk. The major compounds that were identified and quantified by GC–MS were cyanidin 3-glucoside, delphinidin 3-glucoside and pelargonidin 3-glucoside with the percent quantities as 33.23%, 36.54% and 21.32% respectively along with some other minor components presented in trace amounts. Cyanidin-3-glucoside (447 m/z), Delphinidin-3-glucoside (467m/z) and pelargonidin 3-glucoside (463 m/z)

GC-MS was performed by Agilent gas chromatograph (Agilent Technologies, USA) connected with mass Agilent detector (HP-5973 mass) (Ionization energy: 70 eV) using phenylmethylsiloxane capillary column (HHP-5MS 5%). 2mg/ml of the sample was injected using Helium gas (FR 1ml/min) through pre-filter unit. Initially temperature of the column was maintained at 60°C and then increased to 270°C with 10°C/min increase of temperature. Mass spectra were scanned from 10 to 600 u. Comparison were made with previous record and standards. Ratio of the daughter and parents’ ions m/z confirmed the presence of anthocyanins
